# Changes in access to water and incidence of waterborne diseases after the Vale dam collapse in Brumadinho (MG), Brazil

**DOI:** 10.1590/1980-549720230010

**Published:** 2023-01-27

**Authors:** Nayara Trovão, Priscila Neves-Silva, Leticia Cavalari Pinheiro, Sergio Viana Peixoto, Leo Heller

**Affiliations:** I Oswaldo Cruz Foundation, Rene Rachou Institute, Public Policies and Human Rights for Health and Sanitation – Belo Horizonte (MG), Brazil.; II Oswaldo Cruz Foundation, Rene Rachou Institute, Center for Studies in Public Health and Aging – Belo Horizonte (MG), Brazil.; III Oswaldo Cruz Foundation, Rene Rachou Institute, Center for Studies in Public Health and Aging, Nursing School – Belo Horizonte (MG), Brazil.

**Keywords:** Sanitation, Water supply, Human rights, Dam failure, Infectious diseases, Saneamento, Abastecimento de água, Direitos humanos, Rompimento de barragens, Doenças infecciosas

## Abstract

**Objective:**

To describe, within the Human Rights to Water and Sanitation (HRWS) framework, the access to water supply services and the incidence of waterborne diseases in the communities affected by the dam disaster in Brumadinho (MG), Brazil.

**Methods:**

A quantitative and qualitative methodology was used, having as variables information on access to water supply services and waterborne diseases. The primary data were extracted from the “Brumadinho Health Project”, using a sample stratum with 981 people interviewed, totaling 92.5% of the eligible population in the affected communities of Córrego do Feijão and Parque da Cachoeira. The secondary data from Brumadinho was extracted from the project “Sanitation conditions and the River Basin of the B1 River Basin of Mineradora Vale between 2017 and 2020”, available in public databases between 2017 and 2020, and qualitative data was collected in 2022 through individual interviews with health professional also live in the communities.

**Results:**

With regard to access to water supply services, the results of this combined data analysis indicate that the HRWS is being neglected, especially with regard to availability, accessibility, acceptability and quality of water. The study also shows a significant increase in the incidence of waterborne diseases in the region after the disaster.

**Conclusion:**

It is necessary to use the HRWS as the basis to the implementation of public policies aiming to reduce vulnerability in access to water supply services.

## RESUMO

**Objetivo:**

Descrever, na ótica dos direitos humanos à água e ao esgotamento sanitário (DHAES), o acesso à água e a incidência de doenças de veiculação hídrica na região atingida pelo rompimento da barragem da Mina B1 em Brumadinho (MG).

**Métodos:**

Utilizou-se a metodologia quantitativa e qualitativa, tendo como variáveis, em ambos os métodos, informações sobre o acesso à água e as doenças de veiculação hídrica. Os dados primários foram extraídos do Projeto de Saúde Brumadinho, sendo aqui utilizado um estrato amostral com 981 pessoas entrevistadas, totalizando 92,5% da população elegível das comunidades atingidas de Córrego do Feijão e Parque da Cachoeira. Os dados secundários de Brumadinho foram coletados pelo Projeto Condições de Saneamento e Saúde da População da Bacia do Rio Paraopeba, a Jusante da Barragem B1 da Mineradora Vale, entre 2017 e 2020, em banco de dados públicos entre 2017 e 2020, e os dados qualitativos foram coletados em 2022 por meio de entrevistas individuais com profissionais de saúde residentes nas comunidades atingidas.

**Resultados:**

No tocante ao acesso à água, a análise combinada dos dados apontou que os DHAES estão sendo negligenciados na região, de maneira especial no que se refere à disponibilidade, acessibilidade física, aceitabilidade e qualidade da água. Observou-se também que houve aumento significativo na incidência das doenças de veiculação hídrica na região após o desastre.

**Conclusão:**

É necessária utilização dos DHAES como marco referencial na implementação de políticas públicas voltadas para reduzir as situações de vulnerabilidade relacionadas ao acesso à água.

## INTRODUCTION

Access to water of quality and in adequate quantity is a determining factor for promoting health and guaranteeing fundamental human rights such as education, work, health, among others. This understanding led the United Nations (UN) to approve adequate access to water supply and sanitation as a human right in 2010, urging member states to enact laws to ensure access to all in a universal, equal and non-discriminatory manner^1^.

Also according to the UN High Commissioner for Human Rights, access to water supply services, from a human right perspective, must respect requirements such as: availability, quality, acceptability, financial accessibility, and physical accessibility^2^. Therefore, once recognized as a human right, access to water supply becomes an obligation of the State, which must respect, protect and guarantee it. Thus, the State must regulate non-State actors, which is responsible for respecting the human right to water, avoiding any situation that could violate it^3^.

However, when disasters of major proportions occur, such as the rupture of the dam at Mine B1 in Córrego do Feijão (Brumadinho, MG, Brazil) in 2019, administered by the mining company Vale, access to water for the local population is transformed, and new forms of exposure to risks and, consequently, effects on health and local life emerge in the short, medium and long terms.

The collapse of Vale's dam in Brumadinho released around 13 million m^3^ of ore tailings into the environment, which advanced like a gigantic wave over the Paraopeba River basin. This disaster resulted in 265 deaths, five missing people, several injured people and an estimated radius of environmental destruction of 270 hectares on the downstream banks of the Paraopeba River—considering the limits of the municipality of Brumadinho only^4^.

The State Secretariat for the Environment and Sustainable Development, along with the State Health Department and the State Secretariat for Agriculture, Livestock and Supply, recommended that the population do not use raw water from the river for any purpose and determined that the company responsible for the disaster supplied the population with drinking water in safe conditions for its uses.

In the field of health, in addition to causing new risks that compromise access to water supply and water quality, disasters like this can change the cycles of disease vectors, hosts and reservoirs, and overload local health services, impairing their capacity to prevent, detect and care for^5^. Thus, in addition to impairing access to water, such disasters affect the ecosystem, increasing the potential risk of waterborne diseases, offering environmental hazards that will increase morbidity, affecting the future quality of life of the population^6^.

In view of all this, this article aimed to describe access to water supply services and the incidence of waterborne diseases in Córrego do Feijão and in Parque da Cachoeira, from the perspective of the Human Rights to Water and Sanitary (HRWS), after the collapse of the Mina B1 dam in Brumadinho.

## METHODS

This is a quantitative and qualitative study, based on the quantitative data generated by the “Brumadinho Health” Project, which is a prospective cohort study coordinated by the Oswaldo Cruz Foundation in Minas Gerais (Fiocruz Minas) and by Universdiade Federal do Rio de Janeiro, and on the quantitative and qualitative data from the survey “Sanitation and Health Conditions of the Population of the Paraopeba River Basin, Downstream of Mineradora Vale's B1 Dam, between 2017 and 2020”.

### Studied Area

Brumadinho was chosen for this study because it is city where the rupture of the dam at Mina B1 of the mining company Vale happened in 2019. It is located in the Metropolitan Region of Belo Horizonte, and its population data from 2017 to 2020 served as the basis to build the indicators presented here.

The dam was located in the district of Córrego do Feijão and was bordered by the district of Parque da Cachoeira, both belonging to the municipality. These communities were regarded as directly impacted by the disaster.

The sampling plan of the “Brumadinho Health” Project was designed to represent the population aged 12 years or over residing in the municipality and had its first stage carried out in 2021. Specifically for this study, a population cut was made using stratified data from the communities Córrego do Feijão and Parque da Cachoeira. All households in the areas were invited to participate in the survey, corresponding to 1,061 eligible people. The sample had 981 people interviewed in total, accounting for 92.5% of the population of these communities. The 7.5% remaining residents who were not interviewed were refusals, meaning they did not want to participate in the survey or could not be located at their households.

The interviews extracted from the research “Sanitation and Health Conditions of the Population of the Paraopeba River Basin, Downstream of Mineradora Vale's B1 Dam, between 2017 and 2020” were carried out by four health professionals residing in the communities of Córrego do Feijão and Parque da Waterfall.

### Data Collection and Study Variables

The questionnaires in the “Brumadinho Health” Project were built on the basis of previously produced scientific evidence on the effects of disasters on the health of populations. From then on, the interviews were conducted using a structured, standard set of questions, applied by interviewers, with electronic devices, at the participant's residence. The home module was answered by an adult of the household, and the individual interviews were carried out with the resident or with the help of a close respondent, in case the participant had difficulties to answer the questionnaire (which occurred in 9.1% of the interviews).

For analysis purposes of this study, only variables related to “access to water supply” and “waterborne diseases” were selected and arranged in the questionnaire with the following questions: “In the last 30 days, have you had any episodes of colic or abdominal pain?”; “Currently, what is the main form of water supply for this household?”; “Currently, what is the main source of water used for drinking in this household?”.

The secondary data were extracted from the research “Sanitation and Health Conditions of the Population of the Paraopeba River Basin, Downstream of Mineradora Vale's B1 Dam, between 2017 and 2020”, available in public governmental databases National Sanitation Information System (SNIS), Epidemiological Information System of the Department of Informatics of the Unified Health System/Surveillance Information System (Datasus/Sivep), and Automatic Recovery System of the Brazilian Institute of Geography and Statistics (Sidra/IBGE) from 2017 to 2020, regarding the municipality of Brumadinho.

In Sidra/IBGE, the estimated population of Brumadinho for 2017 to 2020 was extracted and served as the basis to calculate the indicators. The analyzed data, taken from SNIS, were about water supply in the municipality in 2017 and 2020. The data taken from the Datasus/Sivep were related to notifications of fecal-oral transmission diseases (acute diarrheal diseases and viral hepatitis) and vector-borne diseases (yellow fever, Dengue, Zika and Chikungunya) for each year. These diseases were chosen because their data are updated in public databases and reflect direct consequences of absence or deficiency in access to water supply on the health of the population.

Qualitative primary data were collected from the same study in March 2022 in interviews using a semi-structured script and aimed at understanding the changes in access to water, as well as in the incidence of waterborne diseases in the communities, from the interviewees’ perspective.

### Data Analysis

The analyses of the data collected by the “Brumadinho Health” Project were carried out in the R software (RCore Team 2021) using the Survey package (Lumley T, 2020), considering sample weight and design effect, necessary when it comes to data from a complex sample. The incidence of variables of interest and respective 95% confidence intervals were estimated, and the Pearson's χ^2^ test with Rao-Scott correction was used to compare the regions of interest.

For the analysis of the secondary data collected in the survey “Sanitation and Health Conditions of the Population of the Paraopeba River Basin, Downstream of Mineradora Vale's B1 Dam, between 2017 and 2020”, the following indicators were established:

- Fecal-oral transmission diseases=(acute diarrheal diseases in reference year+viral hepatitis in reference year)/total population of Brumadinho in reference year;
- Vector-borne diseases (arboviruses)=(reported yellow fever cases in reference year + reported Dengue cases in reference year+reported Zika cases in reference year+reported Chikungunya cases in reference year)/total population of Brumadinho in reference year.

Qualitative data from the same study was assessed by the technique of content analysis^7^.

It should be noted that, in both studies, access to water supply services was analyzed based on the normative elements defined by the HRWS (availability, physical accessibility, quality and acceptability).

### Ethical Considerations

The Brumadinho Health Project was approved by the Research Ethics Committee of Fiocruz Minas (20814719.5.0000.5091). The research “Sanitation and Health Conditions of the Population of the Paraopeba River Basin, Downstream of Mineradora Vale's B1 Dam, between 2017 and 2020” was approved by the Research Ethics Committee of Fiocruz Minas (53250421.0.0000.5091).

In order to maintain confidentiality and preserve the identity of respondents in the survey “Sanitation and Health Conditions of the Population of the Paraopeba River Basin, Downstream of Mineradora Vale's B1 Dam, between 2017 and 2020”, they were identified with the codes E1, E2, E3 and E4, and by acronyms standing for their place of residence: Parque da Cachoeira (PC) and Córrego do Feijão (CF).

In both studies, all participants signed the Free and Informed Consent Form or the Free and Informed Assent Form of the Underaged, accompanied by the Free and Informed Consent Form signed by their guardians.

## RESULTS

### Access to Water Services

According to data from the SNIS, in 2018 only 68.39% of the population of Brumadinho was serviced with water supply (Table 1). Specifically in the PC and CF communities, before the dam rupture, water supply was carried out through reservoirs located in the upper parts of the communities, with water collection directly from the Paraopeba River and/or from local artesian wells^8^. However, even with this form of supply, the availability and accessibility to services were assured, as reported by the interviewees: “A a large artesian well sent water to the houses. I know that the water was never from Copasa [Minas Gerais Sanitation Company] or anything. It was always from that well” (E4, FC); “Before the tragedy, my house did not lack water. There was always plenty of water” (E1, PC).

**Table 1:** Water supply rate in the municipality of Brumadinho (MG), Brazil, between 2017 and 2020.

Reference year	Total population of the municipality	Population served with water supply (%)
2017	38,863	66.83
2018	39,520	68.39
2019	40,103	69.88
2020	40,666	69.55

Source: SIDRA/IBGE (2020), SNIS (2020).

However, the immediate suspension of water from the Paraopeba River after the disaster meant that the reservoirs for collective supply in the affected communities started being supplied by water trucks^9,10^. For 85% of the interviewed in the “Brumadinho Health Project” (Table 2), in 2021, the supply of reservoirs was administered by Copasa, but mining company Vale S.A. provides water by means of water trucks because of the damage repair agreement^9,10^. This is also perceived by local residents: “There is a truck that supplies the water tank now and there is an employee from the city hall who is there doing the treatment” (E4, CF).

**Table 2:** Methods of supplying water to households in Brumadinho (MG), Brazil.

Main form of household water supply	Córrego do Feijão and Parque da Cachoeira* (%)	Others* (%)	p-value
General distribution network (Sanitation Company of Minas Gerais)	85 (83–86)	60.6 (59.4–62)	<0.001
Well or spring	14 (13–15)	36.9 (35.6–38)	
Water truck	0.5 (0.1–2)	2.0 (1.5–3)	
Others	0.5 (0.1–2)	0.5 (0.2–1)	

* Values expressed in percentage and confidence intervals (95%).

Source: Brumadinho Health Project, 2021.

Although data show that accessibility and availability of water are being guaranteed after the dam rupture, the reports of residents of CF and PC communities show that the local reality is different. There has been a recurrent lack of water in the region since the dam collapse, affecting access and availability: “After the tragedy, there is still no water. We lacked water this whole week. Before the tragedy, there was no shortage” (E1, PC).

It is important to emphasize that the guidance given by local authorities to the affected population is to use the collective water supply only for bathing, cleaning the house and other domestic services^10^. So, even with recurrent water supply, the amount available is insufficient to meet the basic needs of the population. In addition, reports show that the little water that reaches the taps is of dubious quality: “We live in suspicion. We take a bath in fear. It has a strong smell coming out. Everything seems to be contaminated: water, soil, vegetables…” (E2, PC). “It is a muddy, yellowish water, which is not normal” (E3, PC).

It should be noted that water for drinking and cooking is provided by the mining company Vale, in accordance with the damage repair agreement, and its continuity is verified in the “Brumadinho Health Project” (Table 3). For residents, at the beginning of the mineral water distribution process shortly after the disaster, in 2019, the amount was sufficient and met family demands, but the weekly distribution was reduced and is now considered insufficient by some who have to fetch water from other places, affecting the concepts of the HRWS related to physical accessibility: “Water lacks here, and Vale is not providing enough. At first, we used to be delivered ten bales; now they deliver five bales every Monday. Just enough to cook, drink, and that is it” (E3, PC).

**Table 3:** Driking water source.

Main source of water used for drinking	Córrego do Feijão and Parque da Cachoeira* (%)	Others* (%)	p-value
General distribution network (Sanitation Company of Minas Gerais)	4.4 (2.8–7)	50.7 (48.4–53)	<0.001
Mineral water	84.7 (82.1–87)	14.7 (12.5–17)	
Others	10.9 (9.4–12)	34.6 (32.9–36)	

* Values expressed in percentage and confidence intervals (95%).

Source: Brumadinho Health Project, 2021.

In the beginning we used to receive 20 bales with six 1.5-liter bottles every week. Now it's down to 10 bales per week. […] When we lack it, we ask [for] someone who lives alone or whose family does not have many people. Those who have more pass it on, helping one another (E4, CF).

It is observed in the reports that water distribution by Vale S.A. increases the distrust of the local population about the contamination of the environment and the Paraopeba River:

It's funny. Vale provides water for consumption, but we shower, water the plants with water not provided by them. So what? I think everyone else thinks the same. Why are they distributing? Because everything is certainly contaminated (E2, PC).

### Diseases Related to Inadequate Water Supply

**Graph 1:**
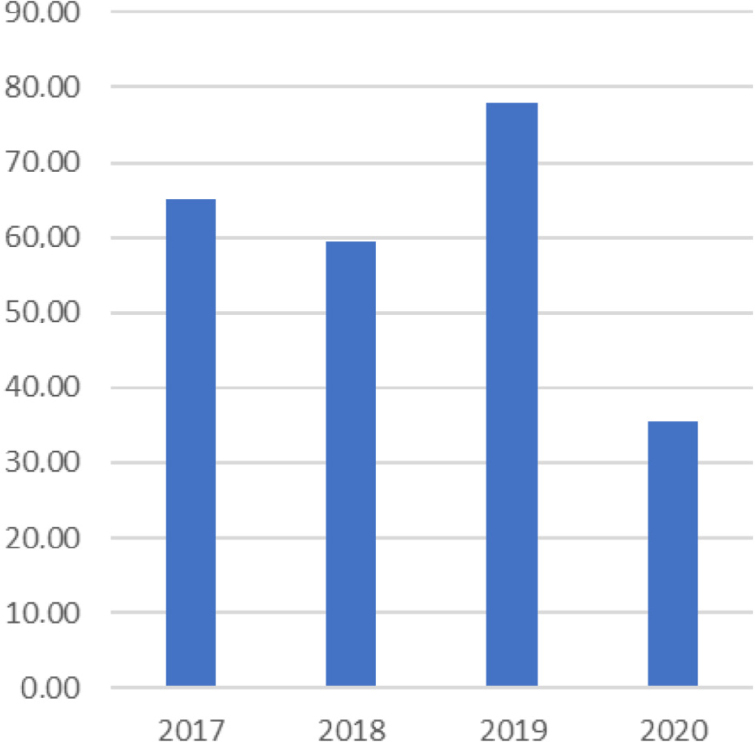
Notification rate of fecal-oral transmitted diseases (per 1,000 inhabitants) in the municipality of Brumadinho (MG), Brazil.

Source: Datasus/Sivep, 2020.

According to the secondary data collected and used by the research “Sanitation and Health Conditions of the Paraopeba River Basin Population, Downstream of Mineradora Vale's B1 Dam, between 2017 and 2020”, in 2019 there was a significant increase in notifications of fecal-oral transmission diseases in relation to pre-disaster years, as shown in Graph 1.

Although these notifications dropped in the municipality in 2020, the “Brumadinho Health Project” identified that in 2021 19% (95% confidence interval — 95%CI: 16.4–22) of the population of CF and PC communities reported some episode of abdominal pain or colic in the past 30 days, against 11.8% (95%CI 10–14) of the population in other regions (p<0.001), indicating that, even with underreporting, the possible symptoms of fecal-oral transmission diseases persist in the population of affected communities.

This underreporting of cases is also mentioned by respondents, associated with intense mistrust about the quality of water that reaches households: “It is very difficult for people to come to the health center because of diarrhea. Unless it's extremely uncomfortable” (E2, PC).

Here, for example, we would pass by a place and someone had diarrhea, then another commented that someone else had it too. All in the same week. Then, the next week, a different group had it. But no one went to the health center. Everything would be treated at home with homemade saline solution and tea (E4, CF).

“Out of six people in the house, only one came to the health unit. So, it means that only one case was notified, while five others were not. Then they say they ate something. It's not that they ate something, it's the water.” (E1, PC).

**Graph 2:**
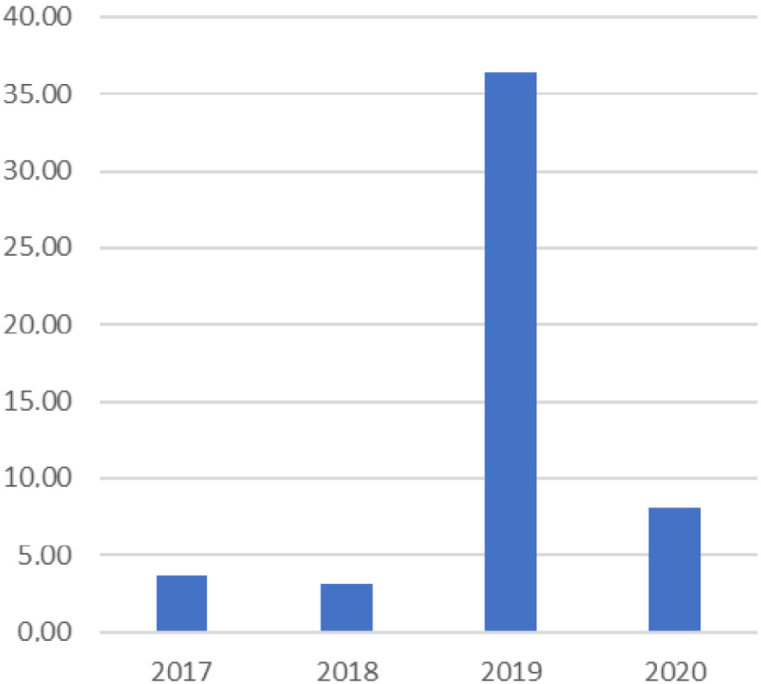
Notification rate of arboviruses (per 1,000 inhabitants) in the municipality of Brumadinho (MG), Brazil.

Source: Datasus/Sivep, 2020.

Secondary data also showed a significant variation in arbovirus notifications (Dengue fever, Zica, yellow fever and Chikungunya) in 2019 compared to previous years, as shown in Graph 2. These diseases should have compulsory notification, but there are signs of possible underreporting: “As soon as the dam collapsed, months passed, there were a lot of cases. But now with these new diseases, Covid-19, sometimes people get Dengue fever and don't even know it. Because nowadays we don't even know how to distinguish between Dengue fever and Covid (E1, PC).

## DISCUSSION

The impacts of large enterprises, such as mining and dams, are driving forces that interfere with the socio-environmental determinants of sanitation^11^. These findings are in line with the impacts identified both in the municipality of Brumadinho as a whole and in the directly impacted communities of CF and PC. The information presented made the relationship between environmental degradation resulting from the dam failure and non-compliance with the normative attributes of the HRWS clear.

According to interviewees, since the dam collapse in early 2019 to the present day, there has been unprecedented water unavailability in the region. Even with the supply of local community reservoirs by water trucks, there is recurrent lack of water for general use.

Regarding the amount of water for drinking and cooking distributed by mining company Vale, it is not considered sufficient by residents, and they depend on the help of neighbors and friends to meet their basic needs. It clear that both the availability and accessibility to water in communities are compromised.

Other than that, the quality of the water that reaches households is questionable, which may be a likely source of waterborne diseases, in addition to basic daily needs not being met, which compromises the achievement of HRWS attribute. This information points to a relationship between the disaster and consequences on the health of the local population.

The worsening in quality of the water that reaches taps, together with the insufficient distribution of drinking water, is reflected by the increase in incidence of waterborne diseases, which was explicit especially in 2019. Studies have widely evidenced the quality and quantity of water as determining factors for acute diarrheal disease^12–19^.

Even with the decrease in cases in the municipality of Brumadinho in 2020 (Graph 1), residents of CF and PC continued to report possible symptoms of fecal-oral transmission diseases both in 2021 and in 2022. This suggests an increase in underreporting of these diseases, as well as temporal continuity of cases in the region.

In the case of arboviruses, they are known to be related to changes in water use, mainly due to water unavailability and accessibility complications. It means that compromised access to water makes the environment favorable for the spread of these diseases^20^. This occurs because the deficiency in water supply forces the population to store water in unprotected reservoirs, without covers or filters, in open air, enabling yet another breeding ground for disease-transmitting mosquitoes^21^.

It should be noted that, for rural populations, the importance of access to water is not limited to issues such as drinking and personal and household hygiene; water is also necessary for the caring of animals, food production for own consumption, income generation and cultural, social and care practices. In this sense, when there is no adequate access to water, these forms of use are impaired, as well as the population's way of life^22,23^.

It can, therefore, be stated that inadequate access to water, in addition to the violation of normative attributes of the HRWS, directly impacts the health of the regions studied, increasing local social inequalities, which may also stress situations of social and economic vulnerability. Therefore, it is necessary to discuss in depth the guarantee of normative attributes in the region, with qualitative and quantitative methodologies, mainly aimed at improving the health of the local population and being an instrument for strengthening the community in the reconstruction of places impacted by the tragedy.
